# Unexpected Mass in the Left Atrium

**DOI:** 10.5935/abc.20180110

**Published:** 2018-08

**Authors:** Tatiana Guimarães, Rui Plácido, Ana Catarina Quadros, José Marques da Costa, Fausto J. Pinto

**Affiliations:** 1 Cardiology Department, Santa Maria University Hospital, CHLN, CAML, CCUL, Faculty of Medicine, University of Lisbon, Lisboa – Portugal; 2 Anatomopathology Department, Santa Maria University Hospital, CHLN, Faculty of Medicine, University of Lisbon, Lisboa – Portugal

**Keywords:** Heart Atria Heart Neoplasms/surgery, Leukemia,Lymphoid/physiopathology, Mitral Valve Stenosis, Echocardiography, Coronary Angiography

A 60-year-old Caucasian female with a history of rheumatic mitral stenosis, permanent atrial fibrillation and chronic lymphocytic leukemia was admitted due to decompensated chronic heart failure. The transthoracic echocardiogram depicted a severe mitral stenosis (anatomic valve area of 0.9 cm^2^), mild mitral regurgitation, aneurysmatic left atrium and mildly compromised left ventricular ejection fraction. Given the indication for mitral valve replacement, coronary angiography was performed, revealing an abnormal vascularized mass at the level of the left atrium beyond normal coronary arteries (Panel A). For better characterization an angio-CT was requested. A well-delimited, 7x4x3cm left atrial homogeneous, slight hyperdense mass was observed along the lateral portion of the atrial roof (Panel B and C). The patient underwent both surgical mass resection and mitral valve replacement with an uneventful recovery. The pathological analysis showed a multifocal left atrial wall and pericardial fat infiltration with CD20+, CD5 +, bcl-2+, cyclin D1+, CD10- and CD23- lymphoid cells, in addition to a left atrial adherent thrombus (Panels D-I*)*. These findings were compatible with lymphocytic lymphoma/chronic lymphocytic leukemia and the patient remains clinically stable. 

Secondary or metastatic tumors are much more common than primary tumors of the heart. A recent necropsy study revealed that cardiac metastases in patients with leukemia and lymphomas may be present in 25% of patients.^1^ Despite being mostly clinically silent, cardiac imaging improvements and availability has led to increased incidental recognition and awareness.

**Figure 1:**
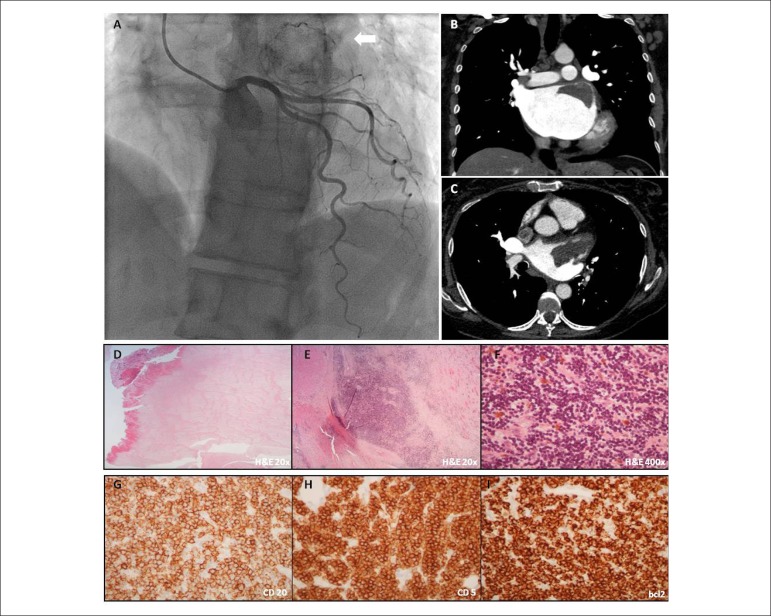
(Panel A) Selective left coronary angiogram (left anterior oblique 30° position) showing an abnormal vascularized mass (arrow) in the left atrium. (Panel B and C) Coronal and axial angio-CT planes in arterial phase, respectively, demonstrating a well-delimited, homogeneous and slight hyperdense mass, along the lateral portion of the atrial roof. (Panel D) Recent thrombus, partially in organization (H&E 20x). (E and F) Myocardium and adipose tissue infiltrated by small lymphoid cells, with scant cytoplasm and nuclei with peripherally clumped chromatin (H&E 20x and 400x). (Panel G-I) CD20, CD5 and bcl2 immunoreactivity (400x), respectively.
